# Short- and long-wavelength lights disrupt endocrine signalling but not immune function in a nocturnal marsupial

**DOI:** 10.1093/conphys/coae092

**Published:** 2025-02-26

**Authors:** Alicia M Dimovski, Kerry V Fanson, Amy M Edwards, Kylie A Robert

**Affiliations:** Department of Animal, Plant and Soil Sciences, School of Agriculture, Biomedicine & Environment, La Trobe University, Bundoora, Victoria 3086, Australia; Research Centre for Future Landscapes, School of Agriculture, Biomedicine & Environment, La Trobe University, Bundoora, Victoria 3086, Australia; Department of Animal, Plant and Soil Sciences, School of Agriculture, Biomedicine & Environment, La Trobe University, Bundoora, Victoria 3086, Australia; Department of Animal, Plant and Soil Sciences, School of Agriculture, Biomedicine & Environment, La Trobe University, Bundoora, Victoria 3086, Australia; Pest and Weeds Unit, New South Wales National Parks and Wildlife Service, Dubbo, New South Wales 2830, Australia; School of Environmental and Rural Science, University of New England, Armidale, New South Wales 2350, Australia; Department of Animal, Plant and Soil Sciences, School of Agriculture, Biomedicine & Environment, La Trobe University, Bundoora, Victoria 3086, Australia; Research Centre for Future Landscapes, School of Agriculture, Biomedicine & Environment, La Trobe University, Bundoora, Victoria 3086, Australia

**Keywords:** Alan, artificial light at night, cortisol, immune function, light pollution, light-emitting diode, melatonin, urbanization

## Abstract

Natural light–dark cycles are responsible for synchronizing an animal’s circadian clock with environmental conditions. Consequently, the endocrine system is vulnerable to changes in the external light environment, particularly short-wavelength blue light. Artificial light at night drastically changes the night-time environment by masking natural light cycles and disrupting well-established biological rhythms. The introduction of blue-rich lighting, such as white light-emitting diodes (LEDs), may increase the biological effects of light at night on wildlife. However, flexibility in the spectral composition of LED lighting presents options for wildlife-sensitive lighting, such as long-wavelength amber LEDs. Here we examine the effect of light spectra on circadian physiology in a nocturnal marsupial. Specifically, we investigate the effect of short-wavelength white (standard urban lighting) and long-wavelength amber LEDs (proposed wildlife-sensitive lighting) on circadian hormones and cell-mediated immunity in the Krefft’s glider (*Petaurus notatus*). Melatonin and glucocorticoid secretion were disrupted following exposure to both short-wavelength white and long-wavelength amber LEDs. Both LEDs suppressed melatonin, whilst glucocorticoid secretion was suppressed under amber LEDs and increased under white LEDs. Despite this disturbance we did not detect any effect of light treatment on cell-mediated immune response. Our findings offer a novel contribution to understanding the physiological impacts of light at night on wildlife. We also provide evidence that long-wavelength amber LEDs can disrupt physiology and are not a wildlife-sensitive lighting option for all species.

## Introduction

Artificial light at night remains one of the most common and fastest growing forms of urban pollution (Linares Arroyo *et al.,* 2024). The introduction of artificial light drastically alters the night-time environment and can disrupt normal photoperiod cues. Light–dark transitions not only entrain daily rhythms, but many animals use change in day length across the seasons to determine time of year (Nelson *et al.,* 1995). Specifically, short-wavelength blue light regulates circadian rhythms and synchronizes animal physiology with seasonal environmental conditions (Bendová and Moravcová, 2019; Grubisic *et al.,* 2019). Artificial light that masks natural day length changes can provide misleading cues and prevent animals from adapting to environmental conditions with negative consequences for survival (Ouyang *et al.,* 2018; Bendová and Moravcová, 2019).

Natural light–dark cycles entrain circadian hormones, including melatonin and glucocorticoids. Light suppresses melatonin, with peak production occurring during the dark phase. In this way melatonin acts as a biological signal for day length (Nelson *et al.,* 1995). Changes in the duration of melatonin secretion throughout the year help synchronize internal biological clocks and regulate seasonal physiological changes, including immune responsiveness (Carrillo-Vico *et al.,* 2005; Bendová and Moravcová, 2019). Melatonin supports the immune system by stimulating production of immune cells and acting as a powerful anti-oxidant (Ahmad *et al.,* 2023). However, exposure to light at night has been shown to disrupt nocturnal melatonin production in invertebrates (Durrant *et al.,* 2015), fish (reviewed in Grubisic *et al.,* 2019 and Yang *et al.,* 2024), birds (reviewed in Grubisic *et al.,* 2019 and Yang *et al.,* 2024) and mammals (Robert *et al.,* 2015; Dimovski and Robert, 2018;Grubisic *et al.,* 2019 ; Yang *et al.,* 2024). The degree of suppression depends on the intensity and spectral characteristics of light (Aubé *et al.,* 2013). The non-image-forming photoreceptors responsible for circadian entrainment are maximally sensitive to blue light (~480 nm; Grubisic *et al.,* 2019). However, sensitivity ranges from 460 to 530 nm and varies between species (Grubisic *et al.,* 2019; Yang *et al.,* 2024). Consequently, blue light affects physiology to a greater extent than orange and red light (Bendová and Moravcová, 2019).

Glucocorticoid secretion is also regulated by the circadian system and peaks at the start of the active period (Chung *et al.,* 2011; Bendová and Moravcová, 2019; Androulakis, 2021). Light stimulates glucocorticoid production in diurnal species and suppresses production in nocturnal species (Androulakis, 2021). Glucocorticoids play an important role in regulating an animal’s response to stressors as well as predictable environmental changes (Romero *et al.,* 2009; Ouyang *et al.,* 2018). Metabolic regulation and long-term energy maintenance are also regulated by glucocorticoids; therefore glucocorticoids can influence fitness (Chung *et al.,* 2011; Romero and Beattie, 2022). Glucocorticoids contribute to synchronizing peripheral clocks with circadian time. Therefore, any disruption to the rhythm or amplitude of glucocorticoid release could disrupt an animal’s physiology. Exposure to pollutants, including artificial light at night, can disrupt the daily rhythm of glucocorticoid synthesis (circadian disruption pathway) or act as a stressor and increase glucocorticoid production via the stress-mediated pathway. (Ouyang *et al.,* 2018).

Melatonin and glucocorticoids play a key role in modulating immune function in mammals (Nelson *et al.,* 1995; Prendergast *et al.,* 2013). Maintaining adequate immune function is critical for surviving challenging conditions; however, investing in immune function is energetically costly, so appropriate timing of this response is critical (Nelson *et al.,* 1995; Lochmiller and Deerenberg, 2000). Regulation of the immune system is complex and modulated by many factors including inflammation, pathogen infection and circadian hormones (Aubrecht *et al.,* 2014). The circadian hormones melatonin and glucocorticoids fluctuate across the year and play a key role in promoting seasonal adjustment of the immune system (Carrillo-Vico *et al.,* 2006), including cell-mediated immune function. Melatonin has immunoenhancing effects (Pandi-Perumal *et al.,* 2006), whilst glucocorticoids can alter the rhythm of the cell-mediated inflammation response (Dhabhar and McEwen, 1999) and chronic stress could suppress the response. Therefore, if artificial light disrupts circadian hormones, animals may also experience a suppressed immune response. If individuals cannot properly anticipate and prepare for harsh environmental conditions, there can be severe fitness consequences at both the individual and population level.

Approximately 80% of marsupials are nocturnal and are therefore amongst the most vulnerable to the disruptive effects of artificial light at night. Previous research in mammals has primarily focused on bats and rodents (e.g. Kramer and Birney, 2001; Tomás-Zapico *et al.,* 2003; Bird *et al.,* 2004; Stone *et al.,* 2009; Bedrosian *et al.,* 2011; Ashkenazi and Haim, 2013; Threlfall *et al.,* 2013). In nocturnal mammals, artificial light delays emergence (Kramer and Birney, 2001; Stone *et al.,* 2009), decreases nocturnal activity (Threlfall *et al.,* 2013), reduces foraging (Bird *et al.,* 2004), delays reproduction (Robert *et al.,* 2015), increases oxidative stress (Tomás-Zapico *et al.,* 2003; Ashkenazi and Haim, 2013) and suppresses immune response (Bedrosian *et al.,* 2011). However, research on the impact of artificial light on the physiological signals controlling daily rhythms and health in wild marsupial mammals is limited, which is particularly concerning given the rapid advancements in lighting technologies.

The introduction of energy-efficient lighting, such as white light-emitting diodes (LEDs), may disrupt the physiology of wildlife to an even greater extent than traditional high-pressure sodium lighting (Ouyang *et al.,* 2018; Bendová and Moravcová, 2019). These white LEDs consist primarily of short, blue wavelengths that regulate circadian rhythms (Grubisic *et al.,* 2019). Whilst the environmental benefits of white LEDs are obvious, there are serious ecological and physiological consequences for wildlife. However, unlike traditional lighting, the spectral composition of LEDs can be manipulated. The use of LEDs that reduce or remove short blue wavelengths, such as amber LEDs, could provide an alternate lighting strategy that is less disruptive to wildlife (Spoelstra *et al.,* 2015; Dimovski and Robert, 2018).

Here we provide the first study to investigate the impacts of different artificial night lighting regimes (spectral wavelengths) on circadian hormones and cell-mediated immunity in an Australian marsupial. Specifically, we aimed to assess (1) melatonin production, (2) glucocorticoid levels and (3) cell-mediated immune function in the Krefft’s glider (*P. notatus*) exposed to ambient night-time conditions and two lighting treatments: short-wavelength white LED lighting (standard urban lighting) and long-wavelength amber LED lighting (proposed wildlife-sensitive lighting). Since short-wavelength, blue light regulates circadian rhythms, the blue-rich white LEDs were predicted to disrupt melatonin, glucocorticoid secretion and cell-mediated immune function to a greater extent than the amber LEDs that do not contain any blue wavelengths. This study enhances our understanding of how artificial light at night affects wildlife physiology and contributes to global standards for wildlife-sensitive lighting.

## Materials and Methods

### Animal capture and housing

Krefft’s gliders (*P. notatus*; previously known as sugar gliders) are a small, nocturnal, omnivorous, arboreal marsupial native to Australia. Krefft’s gliders persist in urban environments making them an ideal model to investigate the effects of artificial light at night on circadian physiology.

This study was conducted from November 2020 to January 2021. We restricted this study to female Krefft’s gliders to prevent aggression between co-housed male gliders from different family groups. Twenty-three wild, female Krefft’s gliders from the same population were collected from pre-existing nest-boxes located in an unlit suburban reserve, the Nangak Tamboree Wildlife Sanctuary (Bundoora, Victoria, Australia) and transferred to large outdoor enclosures (8 m L × 3 m W × 3.5 m H) in the La Trobe University Zoology Reserve (Bundoora, Victoria, Australia). The age (adult/sub-adult) was recorded and all individuals were marked with an implanted chip for individual identity (Trovan ID100 passive integrated transponder tag). Animals were randomly allocated to three treatment enclosures: control (ambient conditions, *n* = 2 adults, 6 sub-adults), amber LEDs (*n* = 2 adults, 6 sub-adults) or white LEDs (*n* = 3 adults, 4 sub-adults). Each enclosure contained individuals captured from different nest-boxes, except for two females in the control and white-treatment groups that were captured from the same nest-box. Enclosures were fitted with natural branches extending from the floor and suspended from the walls and roof of each aviary to provide enrichment and allow animals to freely move throughout the enclosures. Five nest-boxes were provided in each enclosure for animals to shelter and sleep in. Wooden and metal panels were used to block light between enclosures.

Animals were provided food daily before the start of the active period with a nectar mixture comprised of egg, honey, water, vitamin supplement and a high-protein supplement (Wombaroo Food Products, Mount Barker, South Australia, Australia). Animals were also given fresh fruit and vegetables, black fly pupae and mealworm larvae daily. Defrosted day-old chicks were provided once per week. Water was provided *ad libitum* and fresh browse (native foliage and flowering *Eucalyptus* sp., *Acacia* sp. and C*allistemon* sp.) was always available. At the conclusion of the study all animals were released after dark at the point of capture. Approval to conduct this study was granted by the La Trobe University Animal Ethics Committee (AEC 18045) and the Victorian Government Department of Environment, Land, Water and Planning (Permit number 10008921).

### Experimental overview

**Figure 1:**
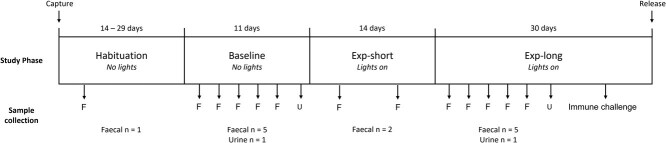
Experimental timeline, number of days during each phase of the experiment and faecal (F) and urine (U) sampling intensity (*n* = number of samples per animal). Faecal samples were collected from animals at the end of the active phase (x- ± SEM = 179 ± 2 min after sunrise). Voluntary urine samples were collected from animals at night, on average (± SEM) 209.7 ± 6.8 minutes after sunset. All immune-challenge pinnae measurements were taken between 08:30 and 10:30 hours.

To investigate the effect of different artificial night lighting regimes (spectral wavelengths) on melatonin production, glucocorticoid secretion and cell-mediated immune function, we captured and temporarily housed 23 wild Krefft’s gliders in large outdoor aviaries for the duration of the study. Animals were habituated to captivity for a period of 14–29 days before experimental procedures commenced (see Fig. 1 for experimental timeline). Following habituation, animals remained under ambient night-time conditions for 11 days whilst baseline levels of urinary melatonin and faecal glucocorticoid metabolites (FGMs) were assessed. Following baseline sampling, animals in the control group remained under ambient night-time conditions, whilst the other groups were exposed to amber or white LEDs at night for 44 days. Faecal samples were collected throughout the study to look at the effect of short and long exposure to light at night on FGM secretion. Night-time urine samples were collected during the baseline and experimental phase to monitor urinary melatonin. Once all experimental urine and faecal samples were collected, delayed-type hypersensitivity (DTH) was conducted to assess cell-mediated immune function.

### Experimental lighting

**Figure 2:**
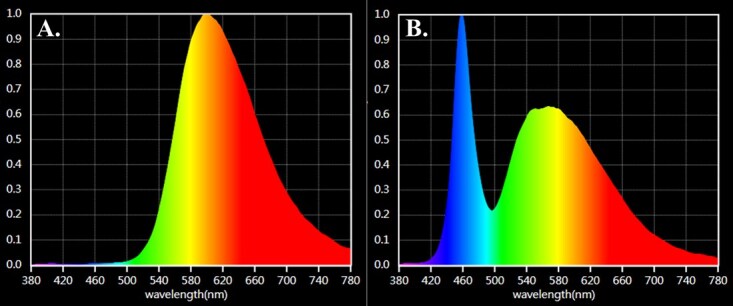
Experimental LED spectra; (A) amber LED (λP 601 nm), (B) cool white LED (λP 460 nm).

Two enclosures were fitted with experimental LED lighting, whilst the third (control enclosure) remained unlit and only exposed to natural night-time conditions throughout the study. Experimental lights were suspended 3 m above the ground in the corner of the enclosure to provide a heterogenous light environment. The average (± standard error of the mean (SEM)) light intensity was 9.79 ± 0.7 lux 1 m from the light source and 3.91 ± 0.14 lux at the back of the enclosure, 4 m from light source in the amber LED treatment, and 11.59 ± 0.63 lux 1 m from the light and 1.49 ± 0.17 lux at the back of the enclosure, 4 m from light source in the white LED treatment. Light intensities at nest-boxes and feed stations are provided in Supplementary Table S2. Light intensities were chosen to reflect the average horizontal illuminance of pedestrian area lighting used in parks and reserves in Australia (PP1; AS/NZS 11:58.3.1:2020). Light intensity remained at 0 lux in the control enclosure throughout the study. Experimental lighting consisted of either amber (λP 601 nm) or white LEDs (λP 460 nm). Light fittings were supplied by Hi-Lux Technical Services Pty Ltd (Thomastown, Victoria, Australia). Lights were connected to digital timers and programmed to turn on and off at sunrise and sunset, respectively (±10 minutes; sunrise and sunset times were obtained from the Australian Bureau of Meteorology; available at http://www.bom.gov.au). A lux meter (EA33 EasyView™ Light Meter with Memory, Extech*)* was used to determine light intensity 1.25–2.25 h after sunset in each experimental enclosure. We quantified the spectral composition of experimental lighting with a handheld spectrometer (Lighting passport, Asensetek, New Taipei, Taiwan; spectral range 380–780 nm; 5–50 000 lux; Fig. 2).

### Urinary melatonin-sulfate analysis

#### Urine sample collection

To assess changes in nocturnal melatonin in response to light treatments, we collected urine samples at two time points: once at the end of the baseline phase and once at the end of the experimental phase once faecal sampling was complete (see Fig. 1 for experimental timeline).

During sampling, animals were removed from nest-boxes at sunset, placed in cotton catch-bags and suspended in the enclosure. During the experimental phase, light intensity inside the catch-bags was 5 lux under amber and white LED lighting (see Supplementary Table S2 for light intensity in enclosures). After 2.5 h (~2300 hours), animals were removed from catch-bags and voided urine was collected. Animals usually urinated upon handling; if required, light touching of the cloaca was used to encourage urination. Voluntary urine samples were collected from animals on average (±SEM) 209.7 ± 6.8 min after sunset. All urine samples were collected in Eppendorf tubes and stored at −20°C until analysis.

#### Urinary melatonin-sulfate enzyme immunoassay

We analysed urine samples for 6-sulfatoxymelatonin (melatonin-sulfate) concentrations, the major melatonin metabolite in urine. Concentrations of urinary melatonin-sulfate correlate with concentrations of circulating melatonin in plasma and saliva (Arendt *et al.,* 1995; Susan *et al.,* 2008). A commercial enzyme-linked immunosorbent assay (Cat no. RE54031; IBL International, Hamburg, Germany) was used to measure melatonin-sulfate in urine samples. The assay was biochemically validated in our lab by demonstrating parallelism between serial dilutions of a pooled urine sample and the standard curve. The parallelism was also used to determine the most appropriate sample dilution for analysis. The assay was run according to the manufacturer’s protocol. Briefly, 50 μl of diluted standard, control or sample was added to the 96-well microtiter plate, followed by 50 μl of enzyme conjugate and 50 μl melatonin-sulfate antiserum. Plates were incubated on an orbital shaker at room temperature for 2 h. Following incubation, plates were washed and loaded with 100 μl TMB substrate and incubated for a further 30 min on an orbital shaker at room temperature. Plates were stopped with 100 μl TMB stop solution and read at 450 nm (620 nm reference filter) using a SPECTROstar Nano plate reader (BMG LABTECH). All samples were run in duplicate and samples from the same individual were run on the same plate. The assay sensitivity was 1.0 pg/ml. Intra-assay coefficients of variation (CVs) were <8.3%. Inter-assay CVs for low and high controls were 5.77 and 3.13% (*n* = 2 plates), respectively.

To account for variation in urine concentration, melatonin-sulfate concentrations are expressed as nanogramme per millilitre creatinine. Creatinine concentration was quantified using a modified Jaffe reaction (described in Fanson *et al.,* 2013). Briefly, 100 μl of standard, control or diluted sample was added to a 96-well microtiter plate, followed by 50 μl of NaOH (0.75 M) and 50 μl picric acid (0.04 M). Plates were incubated for 15 min and read at 450 nm using a SPECTROstar Nano plate reader (BMG LABTECH). Intra-assay CVs were <5.5%. Inter-assay CVs were 2.41 and 1.46% (*n* = 2 plates) for low and high controls, respectively. All samples were run in duplicate and samples from the same individual were run on the same plate.

#### Faecal glucocorticoid metabolite analysis

### Faecal sample collection

Faecal samples were collected from animals 1–3 times per week throughout the three phases of the experiment: baseline, experimental short and experimental long (see Fig. 1 for sampling frequency). During sampling, animals were removed from their nest-box at the end of the active phase (x- ± SEM = 179 ± 2 min after sunrise), and voluntary faecal samples were collected. If animals did not defecate upon handling, they were held in a catch-bag for up to 70 min until a sample was deposited. Bags were checked periodically for faecal samples. Following sample collection, animals were returned to the nest-box and left undisturbed. All samples were immediately stored at −20°C until analysis.

### Steroid extraction

To extract steroid metabolites, 0.02 g (±0.001 g) of wet faecal matter was weighed into individual Eppendorf tubes and 1.0 ml of ethanol (80%) was added. Samples were vortexed and mixed overnight on an orbital shaker (19–21 h). The following day, Eppendorf tubes were centrifuged for 5 min at 2380 RCF and the supernatant was decanted into a clean Eppendorf and stored at −20°C until analysis.

### Faecal glucocorticoid metabolite enzyme immunoassay

FGM concentration was quantified using a cortisol enzyme immunoassay (Arbor Assays, USA, product # ISWE002; see Supplementary Table S1 for validation and assay selection). The assay was run according to manufacturer’s protocol. Briefly, 96-well microtiter plates were coated with goat anti-rabbit IgG (Arbor Assays, USA, A009) and incubated overnight at 4°C. To run the assay, plates were washed and loaded with 50 μl of standard, control or sample, followed by 50 μl of cortisol horseradish peroxidase (HRP) conjugate (1:50) and 50 μl cortisol antibody solution (1:50). Plates were shaken for 2 h at room temperature, then washed and loaded with 100 μl TMB solution. The reaction was stopped after 30 min using 50 μl of stop solution and read at 450 nm (620 nm reference filter) using a SPECTROstar Nano plate reader (BMG LABTECH).

All samples were run in duplicate and randomly allocated across plates. Samples from the same individual were run on the same plate. The EIA was biochemically validated in our lab by demonstrating parallelism between a serially diluted extracted pool and the standard curve. Data is expressed as nanogramme per gramme of wet faeces. Intra-assay CVs were 3.23 and 1.91% (*n* = 16 replicates) for low and high controls, respectively. Inter-assay CVs were 11.49 and 6.44% (*n* = 14 plates), respectively.

### Delayed-type hypersensitivity

DTH was used to analyse changes in cell-mediated immune function following exposure to light at night (see Fig. 1 for experimental timeline). Methods were adapted from Bilbo and Nelson (2003) and Bedrosian *et al.* (2011). Briefly, DTH was induced by sensitization to, and later challenge with, 1-Fluoro-2,4-dinitrobenzene (DNFB, Cat. D1529, Sigma-Aldrich Pty Ltd, Castle Hill, Australia). Animals remained under experimental light at night during the sensitization and challenge phases. Sensitization occurred on Days 1 and 2 by applying 25 μl DNFB (0.5% weight/volume in 4:1 acetone to olive oil vehicle) to a shaved patch on the dorsum. Seven days after sensitization the baseline thickness of both pinnae was measured with a MeasuMax digital outside micrometre (Cat. Q1245, Machinery House, Sydney, Australia). The right pinna was challenged with 20 μl of 0.2% (weight/volume) DNFB in vehicle (4:1 acetone to olive oil). The left pinna was treated with the vehicle solution only. The thickness of the same relative region of both pinnae was measured every 24 h for 5 days. All measurements were made between 0830 and 1030 hours by the same investigator (A.M.D.). Animals were held in catch-bags and manually restrained during sensitization, challenge and measurements. The response to the DNFB challenge reflects cell-mediated immune function.

### Data analysis

All data were analysed using R v4.1.0 (R Development Core Team, 2014).

### Urinary melatonin-sulfate

To examine the effect of light treatment on urinary melatonin-sulfate concentrations, we fitted a linear mixed-effects model using R package ‘lme4’ (Bates *et al.,* 2015) with Satterthwaite approximations for degrees of freedom (R package lmerTest; Kuznetsova *et al.,* 2017). Urinary melatonin-sulfate concentrations were modelled as a function of light treatment (control, amber, white), phase (baseline, experimental) and the interaction of light treatment and phase. Animal ID was included as a random effect to account for repeated samples from individuals. *Post hoc* contrasts were obtained using the emmeans function of the package ‘emmeans’ v1.7.4–1 (Lenth, 2022). Model assumptions of normality and heterogeneity of variance were checked.

### Faecal glucocorticoid metabolites

To examine the effect of light treatment on FGMs, we fitted linear mixed-effects models using R package ‘lme4’ (Bates *et al.,* 2015) with Satterthwaite approximations for degrees of freedom (R package lmerTest; Kuznetsova *et al.,* 2017). FGM concentrations were modelled as a function of light treatment (control, amber, white), phase (baseline, experimental short, experimental long), the interaction of light treatment and phase and animal age (adult, sub-adult). Animal ID was included as a random effect to account for repeated samples from individuals. *Post hoc* contrasts were obtained using the emmeans function of the package ‘emmeans’ v1.7.4–1 (Lenth, 2022). Model assumptions of normality and heterogeneity of variance were checked. FGM concentrations were log-transformed prior to analysis to meet model assumptions.

### Delayed-type hypersensitivity

DTH reactions were calculated as percent increase in pinna thickness over baseline for each animal, corrected for changes in thickness of the non-treated ear (vehicle only). The maximum swelling response was extracted for each individual and modelled as a function of light treatment using a two-way ANOVA (Type III). Model assumptions of normality and heterogeneity of variance were checked. Maximum swelling response were log-transformed prior to analysis to meet model assumptions.

## Results

### Urinary melatonin-sulfate

**Figure 3:**
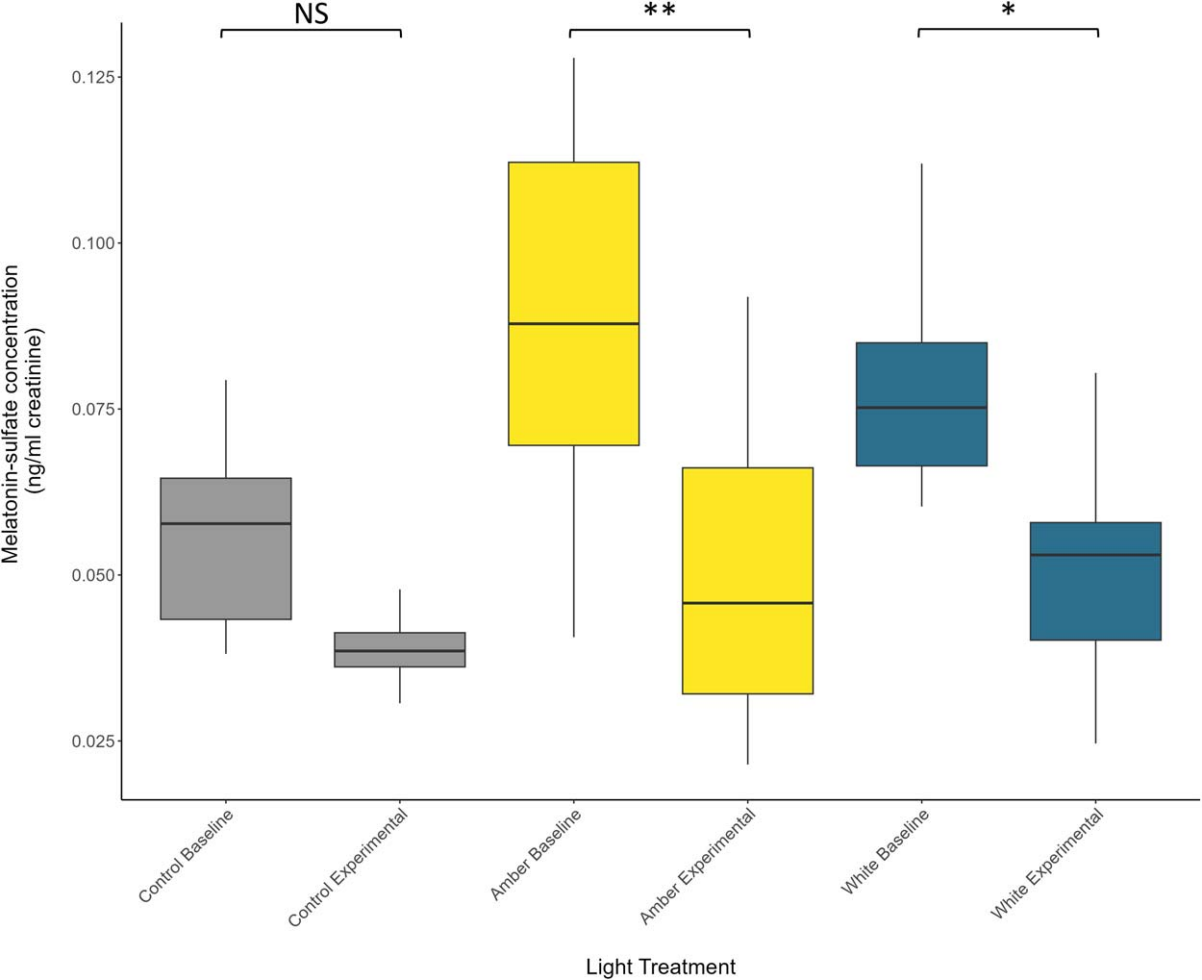
Urinary melatonin-sulfate concentrations (ng/ml creatinine) in Krefft’s gliders (*P. notatus*) exposed to three light treatments: ambient night-time light (control) and two LED light treatments: amber and white. Urinary melatonin-sulfate was measured at two time points: (1) baseline (control *n* = 5, amber *n* = 6; white *n* = 6) and (2) following 28 (±1) days of exposure to experimental light treatments (control *n* = 4; amber *n* = 7; white *n* = 6). Boxes indicate the median value and first and third quartiles, whiskers extend to 1.5 times the interquartile range with outliers beyond. Asterisks represent a significant difference from baseline concentration (^*^P < 0.05, ^**^P < 0.01).

Urinary melatonin-sulfate was significantly higher during the baseline phase compared to the experimental phase (F_1,14.6_ = 14.94, p = 0.002); however we did not detect any significant effect of light treatment or the interaction of light treatment and phase on urinary melatonin-sulfate concentrations (light treatment: F_2,14.99_ = 2.18, *P* = 0.15; light × phase: F_2,14.56_ = 0.56, *P* = 0.58). *Post hoc* analysis revealed phase had a stronger effect for experimental LED treatments than the control. Animals exposed to amber and white LEDs had significantly lower urinary melatonin-sulfate concentrations during the experimental phase compared to the baseline phase (amber: t_14.4_ = 3.2, *P* = 0.006; white: t_13.3_ = 2.4, *P* = 0.03; Fig. 3). In contrast, animals in the control treatment showed no change in melatonin-sulfate concentrations at the experimental phase compared to the baseline phase (t_14.8_ = 1.28, *P* = 0.22; Fig. 3).

### Faecal glucocorticoid metabolites

**Figure 4:**
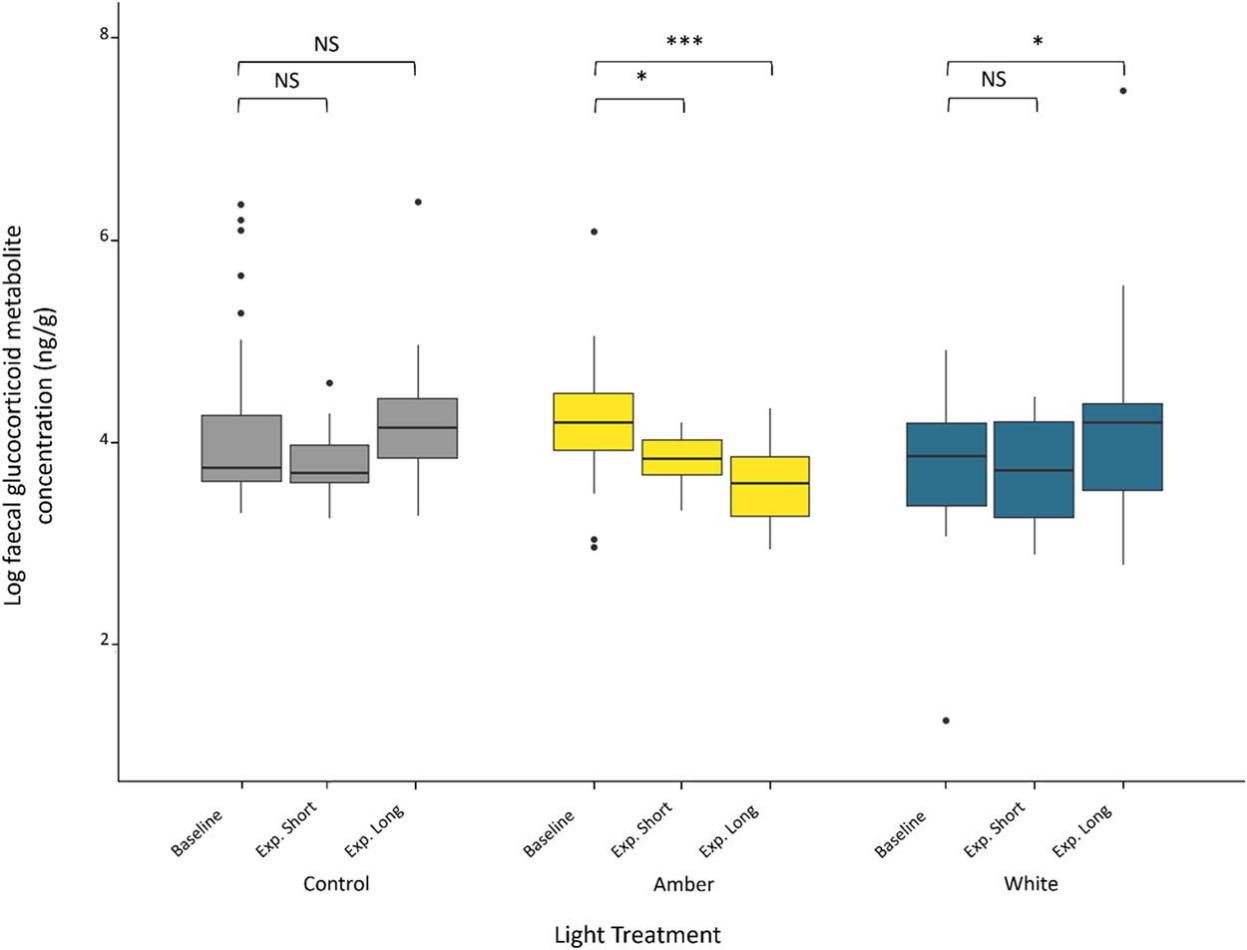
FGM concentrations (ng/g) in Krefft’s gliders (*P. notatus*) exposed to three light treatments: ambient night-time light (control) and two LED light treatments: amber and white. FGM concentrations were determined during three time points: (1) baseline (control *n* = 38; amber *n* = 40; white *n* = 27), (2) following 5–12 days of exposure to experimental light treatments (‘Exp. Short’; control *n* = 15; amber *n* = 16; white *n* = 11) and (3) following 18–26 days of exposure to experimental light treatments (‘Exp. Long’; control *n* = 36; amber *n* = 34; white *n* = 24). Boxes indicate the median value and first and third quartiles, whiskers extend to 1.5 times the interquartile range with outliers beyond. Asterisks represent a significant difference from baseline concentration (^*^P < 0.05, ^***^P < 0.001).

For FGMs, there was a significant interaction between the light treatment and experimental phase (F_4,213.13_ = 7.87, *P* < 0.001; Fig. 4). *Post hoc* analysis showed animals exposed to amber LEDs had lower FGM concentrations at both experimental time points compared to baseline (experimental short: t_213.05_ = −2.47, *P* = 0.03; experimental long: t_217.42_ = −4.6 *P* < 0.001). Under white LED lighting, there was no change in FGMs following experimental short exposure (t_213.12_ = −0.28, *P* = 0.93); however there was a significant increase at the experimental long phase compared to baseline (t_213.76_ = 2.73, *P* = 0.01). There was no change at either time point for animals under ambient night-time (control) conditions (experimental short: t_213.31_ = −0.88, *P* = 0.58; experimental long: t_213.74_ = 1.6, *P* = 0.2). FGMs were significantly higher in adults (x- ± SEM = 178.9 ± 39.2) than sub-adults (x- ± SEM = 56.38 ± 2.67; F_1,17.76_ = 16.89, *P* < 0.1).

**Figure 5:**
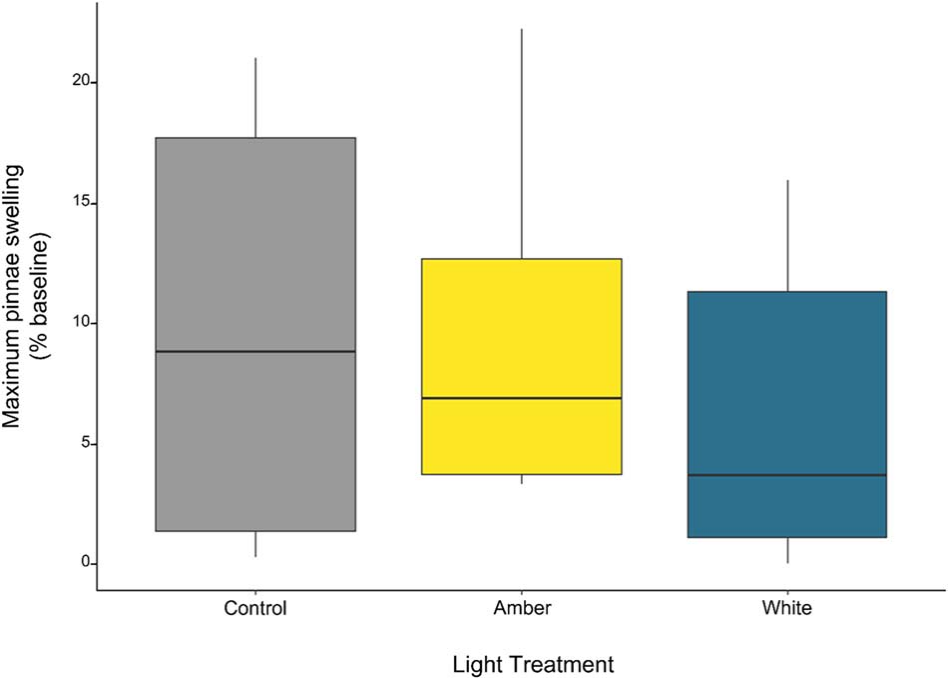
Maximum pinnae swelling (percent increase from baseline) following DTH challenge in Krefft’s gliders (*P. notatus*) following exposure to ambient night-time light (control; *n* = 7) and two LED treatments: amber (*n* = 7), white (*n* = 6). Boxes indicate the median value and first and third quartiles, whiskers extend to 1.5 times the interquartile range with outliers beyond.

### Delayed-type hypersensitivity swelling response

Pinna thickness did not vary in response to light treatment (F_2_ = 0.6, *P* = 0.56; Fig. 5). The mean (±SEM) number of days to peak swelling was 3.43 (±0.48) days in animals in the control treatment, 3.57 (±0.43) days in animals under amber LEDs and 3.83 (±0.6) days in animals exposed to white LEDs.

## Discussion

To our knowledge, this is the first study investigating the impacts of different artificial night lighting regimes (spectral wavelengths) on circadian hormones and cell-mediated immunity in a nocturnal marsupial. We show that in Krefft’s gliders, exposure to amber and white LEDs resulted in significant suppression of urinary melatonin compared to the baseline phase and disrupted glucocorticoid secretion compared to controls. Despite this disturbance, we did not detect any effect of light treatment on cell-mediated immune response.

Consistent with previous studies (Grubisic *et al.,* 2019; Grunst and Grunst, 2023; Yang *et al.,* 2024) we found significant urinary melatonin-sulfate suppression in the Krefft’s glider following exposure to short-wavelength white LEDs. The white LEDs used in our study contain a high proportion of short, blue wavelengths (λP 460 nm, measurable range 420–780 nm), which are expected to have the strongest effect on circadian rhythms. Our findings provide further support for the suppressive effects of blue-wavelength light on melatonin. We also show significant suppression of urinary melatonin-sulfate following exposure to amber LEDs, which do not contain any blue wavelengths (λP 601 nm, measurable range 500–780 nm) and were therefore predicted to be less disruptive. However, the amber LEDs used here still emit light within the circadian sensitivity range (460–530 nm), resulting in the observed urinary melatonin-sulfate suppression. These findings are consistent with previous studies demonstrating a wavelength-dependent response of melatonin (Brainard *et al.,* 1984; Zubidat *et al.,* 2011; Dimovski and Robert, 2018). However, due to our relatively small sample size, further investigation is needed into the effects of light spectra, as well as the timing, duration and intensity of light on urinary melatonin.

Our findings demonstrate circadian disruption of melatonin occurs following exposure to low-intensity short- and long-wavelength LEDs in a nocturnal marsupial. The observed melatonin suppression could result from a reduction in the total nightly volume of melatonin produced, or a shift in the timing of the melatonin peak. To determine which type of suppression is occurring, studies are required to identify circadian patterns of melatonin secretion, develop melatonin suppression curves for non-human species and monitor changes to melatonin rhythms following exposure to light at night. Further work is also needed to identify the long-term health and fitness consequences of the observed melatonin suppression.

Here we demonstrate that night-time exposure to both short-wavelength white LEDs and long-wavelength amber LEDs disrupts glucocorticoid secretion in a nocturnal marsupial. However, the direction of response was different for the two light treatments. Gliders exposed to white LEDs showed no change in glucocorticoid secretion following short exposure and an increase following long exposure. Exposure to artificial light at night has been shown to increase glucocorticoid secretion in a range of taxa including humans (Figueiro and Rea, 2010; Mohawk *et al.,* 2012; Wilson and Downs, 2015), rodents (Bedrosian *et al.,* 2011, 2013b), birds (Ouyang *et al.,* 2015; Russ *et al.,* 2015; Alaasam *et al.,* 2018; reviewed in Grunst and Grunst, 2023) and fish (Migaud *et al.,* 2007). The observed increase in FGMs suggests white LEDs could be masking the difference between day and night (Martinez-Nicolas *et al.,* 2014), thereby disrupting circadian regulation of glucocorticoids in the Krefft’s glider. Alternatively, the observed increase may result from the stress-mediated pathway. Nocturnal mammals decrease their activity under low-intensity light during a full moon and under artificial lights in response to an increased predation risk (Wolfe and Tan Summerlin, 1989; Daly *et al.,* 1992; Vasquez, 1994; Falkenberg and Clarke, 1998; Shier *et al.,* 2020). Consequently, the observed increase in glucocorticoids under white LEDs could be due to the perceived increase in predation risk, resulting in increased physiological stress.

In contrast, FGM concentrations were significantly lower following both short and long exposure to amber LEDs, which do not contain any blue wavelengths (λP 601 nm, measurable range 500–780 nm) and were predicted to be less disruptive. However, these amber LEDs still emit light within the circadian sensitivity range (460–530 nm), resulting in the observed glucocorticoid suppression. The observed decrease in FGMs could result from disrupted circadian regulation of glucocorticoid secretion. Monitoring FGMs provides a pooled measure of adrenal activity. As such, the samples collected here likely reflect adrenal activity late in the active phase. Consequently, suppressed glucocorticoid secretion could have resulted from either an earlier decline in glucocorticoid synthesis at the end of the active phase or a dampening of the daily glucocorticoid rhythm. Similar glucocorticoid suppression has been observed in the striped mouse following 3 days of exposure to intermittent light at night (Wilson and Downs, 2015). Additionally, low-intensity (5 lux) light at night has been shown to blunt diurnal glucocorticoid fluctuations in Siberian hamsters (Bedrosian *et al.,* 2013b). In birds, artificial light has been shown to suppress baseline glucocorticoids whilst increasing acute glucocorticoid production (Injaian *et al.,* 2021). This suggests that exposure to artificial light may differentially affect various components of the hypothalamic–pituitary–adrenal (HPA) axis.

Chronic dysregulation of glucocorticoids, irrespective of the direction of effect, can result in diverse pathological conditions (Chung *et al.,* 2011) and reduced survival (Bradley *et al.,* 1975). Here we demonstrate that exposure to short- and long-wavelength artificial lights disrupt glucocorticoid secretion compared to the control. Chronic increases in glucocorticoids suppress the immune response in hamsters (Bedrosian *et al.,* 2011, 2013b) and are associated with changes in body condition, and parasite and bacterial infection in marsupials (Kemper *et al.,* 1989). Prolonged high glucocorticoids also disrupt homeostasis (Romero and Beattie, 2022) and can increase the rate of oxidative stress and oxidative damage associated with accelerated senescence (Haussmann and Marchetto, 2010; Costantini *et al.,* 2011; Angelier *et al.,* 2018). However, suppressed glucocorticoid secretion does not necessarily indicate a good physiological condition and can result in lack of adequate coping to environmental changes (Schoenle *et al.,* 2018; Romero and Beattie, 2022). Disrupted glucocorticoid secretion can disrupt synchronization of peripheral clocks with physiological consequences observed throughout the body (Bendová and Moravcová, 2019). Despite numerous studies demonstrating light at night disrupts glucocorticoid secretion, the long-term fitness and reproductive consequences in wild mammals remain largely unknown (Ouyang *et al.,* 2018). Future studies are required to determine the circadian pattern of glucocorticoid release and investigate whether exposure to artificial lighting alters the timing of glucocorticoid peaks or the daily rhythm of glucocorticoids. Additionally, long-term consequences of disrupted glucocorticoid secretion on reproduction, disease and survival are required to evaluate the effect of light pollution on wild populations.

We used a DTH challenge to assess cell-mediated immunity following 5 weeks of light at night. We did not observe any effect of light treatment on inflammation response. Our findings conflict with previous research showing dim light at night suppresses cell-mediated immune response in Siberian hamsters (Bedrosian *et al.,* 2011; Aubrecht *et al.,* 2014), suggesting the immune system is sensitive to low levels of light at night. In contrast, dim light at night increases swelling response in the diurnal grass rat (Fonken *et al.,* 2012). However not all studies have demonstrated a change to the immune response following exposure to light at night. Siberian hamsters exposed to 8 weeks of dim light at night showed no change in swelling response (Ikeno *et al.,* 2014). We conducted our study during long days, which may have dampened the response observed. Additionally, we used outdoor enclosures where animals in the control treatment were exposed to natural night-time conditions, including lunar illumination, in contrast to lab studies, which use complete darkness for control conditions. Unnaturally dark conditions may exacerbate the difference observed in response of control and light-exposed groups.

The DTH response reflects T cell-mediated immunity, which is sensitive to melatonin and glucocorticoids (Fonken *et al.,* 2012; Bedrosian *et al.,* 2013a; Aubrecht *et al.,* 2014; Ikeno *et al.,* 2014). Despite observing disrupted circadian hormones, we did not detect any changes in cell-mediated immunity. However, different components of the immune system may be differentially affected by light at night. Further research is required into the immune–pineal axis and the immune–HPA axis to identify long-term consequences of disrupted circadian hormones on immunity and survival.

Investigating the effect of artificial light at night on circadian physiology and immunity is critical to identify appropriate lighting options for wildlife-sensitive areas. Here we demonstrate that both short-wavelength white and long-wavelength amber LEDs disrupt circadian hormone secretion in a nocturnal marsupial. Consequently, amber LEDs cannot be considered a wildlife-sensitive lighting option for all species. Future research is needed to explore the effects of artificial light on daily melatonin and glucocorticoid rhythms and identify the long-term impacts of this disturbance. Our findings contribute novel insights into the physiological impacts of light at night on wildlife and show that changing the colour of LEDs is not an effective management strategy for all species.

## Supplementary Material

Web_Material_coae092

## Data Availability

The dataset on which this article is based is available from Figshare data repository at doi:10.26181/23692986.

## References

[ref1] Ahmad SB, Ali A, Bilal M, Rashid SM, Wani AB, Bhat RR, Rehman MU (2023) Melatonin and health: insights of melatonin action, biological functions, and associated disorders. Cell Mol Neurobiol 43: 2437–2458. 10.1007/s10571-023-01324-w. 36752886 PMC9907215

[ref2] Alaasam VJ, Duncan R, Casagrande S, Davies S, Sidher A, Seymoure B, Shen Y, Zhang Y, Ouyang JQ (2018) Light at night disrupts nocturnal rest and elevates glucocorticoids at cool color temperatures. J Exp Zool A Ecol Integr Physiol 329: 465–472. 10.1002/jez.2168. 29766666 PMC6205889

[ref3] Androulakis IP (2021) Circadian rhythms and the HPA axis: a systems view. Wiley Interdiscip Rev Mech Dis 13: e1518. 10.1002/wsbm.1518. PMC8900069 33438348

[ref4] Angelier F, Costantini D, Blévin P, Chastel O (2018) Do glucocorticoids mediate the link between environmental conditions and telomere dynamics in wild vertebrates? A review. Gen Comp Endocrinol 256: 99–111. 10.1016/j.ygcen.2017.07.007. 28705731

[ref5] Arendt J, Deacon S, English J, Hampton S, Morgan L (1995) Melatonin and adjustment to phase shift. J Sleep Res 4: 74–79. 10.1111/j.1365-2869.1995.tb00232.x. 10607217

[ref6] Ashkenazi L, Haim A (2013) Effect of light at night on oxidative stress markers in golden spiny mice (Acomys russatus) liver. Comp Biochem Physiol A Mol Integr Physiol 165: 353–357. 10.1016/j.cbpa.2013.04.013. 23608365

[ref7] Aubé M, Roby J, Kocifaj M (2013) Evaluating potential spectral impacts of various artificial lights on melatonin suppression, photosynthesis, and star visibility. PloS One 8: e67798. 10.1371/journal.pone.0067798. 23861808 PMC3702543

[ref8] Aubrecht TG, Weil ZM, Nelson RJ (2014) Dim light at night interferes with the development of the short-day phenotype and impairs cell-mediated immunity in Siberian hamsters (*Phodopus sungorus*). J Exp Zool A Ecol Genet Physiol 321: 450–456. 10.1002/jez.1877. 24962267

[ref9] Bates D, Mächler M, Zurich E, Bolker BM, Walker SC (2015) Fitting linear mixed-effects models using lme4. J Stat Softw 67: 1–48. 10.18637/jss.v067.i01.

[ref10] Bedrosian T, Aubrecht TG, Kaugars KE, Weil ZM, Nelson RJ (2013a) Artificial light at night alters delayed-type hypersensitivity reaction in response to acute stress in Siberian hamsters. Brain Behav Immun 34: 39–42. 10.1016/j.bbi.2013.05.009. 23743259

[ref11] Bedrosian T, Galan A, Vaughn CA, Weil ZM, Nelson RJ (2013b) Light at night alters daily patterns of cortisol and clock proteins in female Siberian hamsters. J Neuroendocrinol 25: 590–596. 10.1111/jne.12036. 23489976

[ref12] Bedrosian TA, Fonken LK, Walton JC, Nelson RJ (2011) Chronic exposure to dim light at night suppresses immune responses in Siberian hamsters. Biol Lett 7: 468–471. 10.1098/rsbl.2010.1108. 21270021 PMC3097873

[ref13] Bendová Z, Moravcová S (2018) Erasing day/night differences in light intensity and spectrum affect biodiversity and the health of mammals by confusing the circadian clock. Lynx (Praha) 49: 139–161. 10.2478/lynx-2018-0012.

[ref14] Bilbo SD, Nelson RJ (2003) Sex differences in photoperiodic and stress-induced enhancement of immune function in Siberian hamsters. Brain Behav Immun 17: 462–472. 10.1016/S0889-1591(03)00063-1. 14583238

[ref15] Bird BL, Branch LC, Miller DL (2004) Effects of coastal lighting on foraging behavior of beach mice. Conserv Biol 18: 1435–1439. 10.1111/j.1523-1739.2004.00349.x.

[ref16] Bradley AJ, McDonald IR, Lee AK (1975) Effect of exogenous cortisol on mortality of a dasyurid marsupial. J Endocrinol 66: 281–282. 10.1677/joe.0.0660281. 1165455

[ref17] Brainard GC, Richardson BA, King TS, Reiter RJ (1984) The influence of different light spectra on the suppression of pineal melatonin content in the Syrian hamster. Brain Res 294: 333–339. 10.1016/0006-8993(84)91045-X. 6704731

[ref18] Carrillo-Vico A, Guerrero JM, Lardone PJ, Reiter RJ (2005) A review of the multiple actions of melatonin on the immune system. Endocrine 27: 189–200. 10.1385/ENDO:27:2:189. 16217132

[ref19] Carrillo-Vico A, Reiter RJ, Lardone PJ, Herrera JL, Fernández-Montesinos R, Guerrero JM, Pozo D (2006) The modulatory role of melatonin on immune responsiveness. Curr Opin Investig Drugs 7: 423–431. 16729718

[ref20] Chung S, Son GH, Kim K (2011) Circadian rhythm of adrenal glucocorticoid: its regulation and clinical implications. Biochimica et Biophysica Acta (BBA) - Molecular Basis of Disease 1812: 581–591. 10.1016/j.bbadis.2011.02.003. 21320597

[ref21] Costantini D, Marasco V, Møller AP (2011) A meta-analysis of glucocorticoids as modulators of oxidative stress in vertebrates. J Comp Physiol B 181: 447–456. 10.1007/s00360-011-0566-2. 21416253

[ref22] Daly M, Behrends PR, Wilson MI, Jacobs LF (1992) Behavioural modulation of predation risk: moonlight avoidance and crepuscular compensation in a nocturnal desert rodent, Dipodomys merriami. Anim Behav 44: 1–9. 10.1016/S0003-3472(05)80748-1.

[ref23] Dhabhar FS, McEwen BS (1999) Enhancing versus suppressive effects of stress hormones on skin immune function. Proc Natl Acad Sci U S A 96: 1059–1064. 10.1073/pnas.96.3.1059. 9927693 PMC15350

[ref24] Dimovski AM, Robert KA (2018) Artificial light pollution: shifting spectral wavelengths to mitigate physiological and health consequences in a nocturnal marsupial mammal. J Exp Zool A Ecol Integr Physiol 329: 497–505. 10.1002/jez.2163. 29722167

[ref25] Durrant J, Michaelides EB, Rupasinghe T, Tull D, Green MP, Jones TM (2015) Constant illumination reduces circulating melatonin and impairs immune function in the cricket Teleogryllus commodus. PeerJ 3: e1075. 10.7717/peerj.1075. 26339535 PMC4558066

[ref26] Falkenberg JC, Clarke JA (1998) Microhabitat use of deer mice: effects of interspecific interaction risks. J Mammal 79: 558–565. 10.2307/1382986.

[ref27] Fanson K, Lynch M, Vogelnest L, Miller G, Keeley T (2013) Response to long-distance relocation in Asian elephants (*Elephas maximus*): monitoring adrenocortical activity via serum, urine, and feces. Eur J Wildl Res 59: 655–664. 10.1007/s10344-013-0718-7.

[ref28] Figueiro MG, Rea MS (2010) The effects of red and blue lights on circadian variations in cortisol, alpha amylase, and melatonin. Int J Endocrinol 2010: 829351. 20652045 10.1155/2010/829351 PMC2905913

[ref29] Fonken LK, Haim A, Nelson RJ (2012) Dim light at night increases immune function in Nile grass rats, a diurnal rodent. Chronobiol Int 29: 26–34. 10.3109/07420528.2011.635831. 22217098

[ref30] Grubisic M, Haim A, Bhusal P, Dominoni DM, Gabriel KMA, Jechow A, Kupprat F, Lerner A, Marchant P, Riley W et al. (2019) Light pollution, circadian photoreception, and melatonin in vertebrates. Sustainability 11: 6400. 10.3390/su11226400.

[ref31] Grunst ML, Grunst AS (2023) Endocrine effects of exposure to artificial light at night: a review and synthesis of knowledge gaps. Mol Cell Endocrinol 568–569: 111927. 10.1016/j.mce.2023.111927 37019171

[ref32] Haussmann MF, Marchetto NM (2010) Telomeres: linking stress and survival, ecology and evolution. Curr Zool 56: 714–727. 10.1093/czoolo/56.6.714.

[ref33] Ikeno T, Weil ZM, Nelson RJ (2014) Dim light at night disrupts the short-day response in Siberian hamsters. Gen Comp Endocrinol 197: 56–64. 10.1016/j.ygcen.2013.12.005. 24362257

[ref34] Injaian AS, Uehling JJ, Taff CC, Vitousek MN (2021) Effects of artificial light at night on avian provisioning, corticosterone, and reproductive success. Integr Comp Biol 61: 1147–1159. 10.1093/icb/icab055. 34021748

[ref35] Kemper C, Kitchener DJ, Humphreys WF, How RA, Schmitt LH, Bradley A (1989) The biology of the northern brown bandicoot, Isoodon-Macrourus (Marsupialia, Peramelidae) at Mitchell Plateau, Western-Australia. Aust J Zool 37: 627–644. 10.1071/ZO9890627.

[ref36] Kramer KM, Birney EC (2001) Effect of light intensity on activity patterns of Patagonian leaf-eared mice, *Phyllotis xanthopygus*. J Mammal 82: 535–544. 10.1644/1545-1542(2001)082<0535:EOLIOA>2.0.CO;2.

[ref37] Kuznetsova A, Brockhoff PB, Christensen RHB (2017) lmerTest package: tests in linear mixed effects models. J Stat Softw 82: 1–26. 10.18637/jss.v082.i13.

[ref38] Lenth RV (2022) Estimated marginal means, aka least-squares means. R package version 1.10.1.

[ref39] Linares Arroyo H, Abascal A, Degen T, Aubé M, Espey BR, Gyuk G, Hölker F, Jechow A, Kuffer M, Sánchez de Miguel A et al. (2024) Monitoring, trends and impacts of light pollution. Nat Rev Earth Environ 5: 417–430. 10.1038/s43017-024-00555-9.

[ref40] Lochmiller RL, Deerenberg C (2000) Trade-offs in evolutionary immunology: just what is the cost of immunity? Oikos 88: 87–98. 10.1034/j.1600-0706.2000.880110.x.

[ref41] Martinez-Nicolas A, Madrid JA, Rol MA (2014) Day–night contrast as source of health for the human circadian system. Chronobiol Int 31: 382–393. 10.3109/07420528.2013.861845. 24304407

[ref42] Migaud H, Cowan M, Taylor J, Ferguson HW (2007) The effect of spectral composition and light intensity on melatonin, stress and retinal damage in post-smolt Atlantic salmon, Salmo salar. Aquaculture 270: 390–404. 10.1016/j.aquaculture.2007.04.064.

[ref43] Mohawk JA, Green CB, Takahashi JS (2012) Central and peripheral circadian clocks in mammals. Annu Rev Neurosci 35: 445–462. 10.1146/annurev-neuro-060909-153128. 22483041 PMC3710582

[ref44] Nelson RJ, Demas GE, Klein SL, Kriegsfeld LJ (1995) Minireview the influence of season, photoperiod, and pineal melatonin on immune function. J Pineal Res 19: 149–165. 10.1111/j.1600-079X.1995.tb00184.x. 8789246 PMC7166827

[ref45] Ouyang JQ, Davies S, Dominoni D (2018) Hormonally mediated effects of artificial light at night on behavior and fitness : linking endocrine mechanisms with function. J Exp Biol 221: jeb 156893. 10.1242/jeb.156893. PMC5897701 29545373

[ref46] Ouyang JQ, de Jong M, Hau M, Visser ME, van Grunsven RHA, Spoelstra K (2015) Stressful colours: corticosterone concentrations in a free-living songbird vary with the spectral composition of experimental illumination. Biol Lett 11: 20150517. 10.1098/rsbl.2015.0517. 26311159 PMC4571683

[ref47] Pandi-Perumal SR, Srinivasan V, Maestroni GJM, Cardinali DP, Poeggeler B, Hardeland R (2006) Melatonin: nature’s most versatile biological signal? FEBS Journal 273: 2813–2838. 10.1111/j.1742-4658.2006.05322.x. 16817850

[ref48] Prendergast BJ, Cable EJ, Patel PN, Pyter LM, Onishi KG, Stevenson TJ, Ruby NF, Bradley SP (2013) Impaired leukocyte trafficking and skin inflammatory responses in hamsters lacking a functional circadian system. Brain Behav Immun 32: 94–104. 10.1016/j.bbi.2013.02.007. 23474187 PMC3686870

[ref49] R Development Core Team (2014) R: A Language and Environment for Statistical Computing. R Foundation for Statistical Computing, Vienna, Austria.

[ref50] Robert KA, Lesku JA, Partecke J, Chambers B (2015) Artificial light at night desynchronizes strictly seasonal reproduction in a wild mammal. Proc R Soc B 282: 20151745. 10.1098/rspb.2015.1745. PMC4614780 26423847

[ref51] Romero LM, Beattie UK (2022) Common myths of glucocorticoid function in ecology and conservation. J Exp Zool A Ecol Integr Physiol 337: 7–14. 10.1002/jez.2459. 33819389

[ref52] Romero LM, Dickens MJ, Cyr NE (2009) The reactive scope model — a new model integrating homeostasis, allostasis, and stress. Horm Behav 55: 375–389. 10.1016/j.yhbeh.2008.12.009. 19470371

[ref53] Russ A, Reitemeier S, Weissmann A, Gottschalk J, Einspanier A, Klenke R (2015) Seasonal and urban effects on the endocrinology of a wild passerine. Ecol Evol 5: 5698–5710. 10.1002/ece3.1820. 27069618 PMC4813110

[ref54] Schoenle LA, Zimmer C, Vitousek MN (2018) Understanding context dependence in glucocorticoid–fitness relationships: the role of the nature of the challenge, the intensity and frequency of stressors, and life history. Integr Comp Biol 58: 777–789. 10.1093/icb/icy046. 29889246

[ref55] Shier DM, Bird AK, Wang TB (2020) Effects of artificial light at night on the foraging behavior of an endangered nocturnal mammal. Environ Pollut 263: 114566. 10.1016/j.envpol.2020.114566. 32320890

[ref56] Spoelstra K, Van Grunsven RHA, Donners M, Gienapp P, Huigens ME, Slaterus R, Berendse F, Visser ME, Veenendaal E (2015) Experimental illumination of natural habitat—an experimental set-up to assess the direct and indirect ecological consequences of artificial light of different spectral composition. Philos Trans R Soc B 370: 20140129. 10.1098/rstb.2014.0129. PMC4375369 25780241

[ref57] Stone EL, Jones G, Harris S (2009) Street lighting disturbs commuting bats. Curr Biol 19: 1123–1127. 10.1016/j.cub.2009.05.058. 19540116

[ref58] Susan B, Burgess H, Elizabeth K, Alfred L, Benita M, Patricia M, Barbara P, Victoria R (2008) Measuring melatonin in humans. J Clin Sleep Med 4: 66–69. 10.5664/jcsm.27083. 18350967 PMC2276833

[ref59] Threlfall CG, Law B, Banks PB (2013) The urban matrix and artificial light restricts the nightly ranging behaviour of Gould’s long-eared bat (Nyctophilus gouldi). Austral Ecol 38: 921–930. 10.1111/aec.12034.

[ref60] Tomás-Zapico C, Coto-Montes A, Martínez-Fraga J, Rodríguez-Colunga MJ, Tolivia D (2003) Effects of continuous light exposure on antioxidant enzymes, porphyric enzymes and cellular damage in the Harderian gland of the Syrian hamster. J Pineal Res 34: 60–68. 10.1034/j.1600-079X.2003.02951.x. 12485373

[ref61] Vasquez RA (1994) Assessment of predation risk via illumination level: facultative central place foraging in the cricetid rodent *Phyllotis darwini*. Behav Ecol Sociobiol 34: 375–381. 10.1007/BF00197008.

[ref62] Wilson AL, Downs CT (2015) Light interference and melatonin affects digestion and glucocorticoid metabolites in striped mouse. Biol Rhythm Res 46: 929–939. 10.1080/09291016.2015.1066546.

[ref63] Wolfe JL, Tan Summerlin C (1989) The influence of lunar light on nocturnal activity of the old-field mouse. Anim Behav 37: 410–414. 10.1016/0003-3472(89)90088-2.

[ref64] Yang Y, Liu Q, Pan C, Chen J, Xu B, Liu K, Pan J, Lagisz M, Nakagawa S (2024) Species sensitivities to artificial light at night: a phylogenetically controlled multilevel meta-analysis on melatonin suppression. Ecol Lett 27: e14387. 10.1111/ele.14387. 38382914

[ref65] Zubidat AE, Nelson RJ, Haim A (2011) Spectral and duration sensitivity to light-at-night in ‘blind’ and sighted rodent species. J Exp Biol 214: 3206–3217. 10.1242/jeb.058883. 21900468

